# The pre-surgical role of halo-traction in patients with cervical infection associated with refractory kyphosis: a retrospective study

**DOI:** 10.1038/s41598-023-27523-5

**Published:** 2023-01-10

**Authors:** Daudi R. Manini, Hong-Qi Zhang, Qile Gao, Shao-Hua Liu, Wang YuXiang, Ming-Xing Tang, Deng An, Chao-Feng Guo, Du YuXuan

**Affiliations:** 1 Department of Spine Surgery and Orthopaedics, Xiangya Hospital, Central South University, Changsha, 410008 China; 2 National Clinical Research Center for Geriatric Disorders, Xiangya Hospital, Central South University, Xiangya Road 87, Changsha, 410008 China

**Keywords:** Diseases, Medical research, Neurology

## Abstract

To minimize surgical complications and staged procedures halo-traction is often used during deformity corrections. But the use of halo-traction in the treatment of refractory cervical kyphosis secondary to infections has never been reported. This study investigated the role of halo-traction in the treatment of cervical infection patients associated with refractory kyphosis. We retrospectively reviewed 48 patients with cervical infection associated with refractory kyphosis who were treated in our spine department. Patients were divided into two groups, the traction group (A) and the non-traction group (B). Group A underwent preoperative halo-traction followed by surgery, while group B underwent surgery alone. Between the two groups, we analyzed the kyphosis deformity correction, level of fusions, erythrocyte sedimentation rate (ESR), C-reactive protein (CRP), functional improvement by Neck disability index (NDI) score, and complications. Group A had a better correction of kyphosis deformity compared to group B (27.01 ± 11.54)^0^ versus (18.08 ± 10.04)^0^ (P = 0.01, Z =  − 2.44). No statistically significant differences between the two groups in terms of functional improvement, level of fusions, ESR and CRP. Group B had 3 revision surgery cases. Preoperative halo-traction followed by surgery is superior in kyphosis correction in the treatment of patients with cervical infections with refractory kyphosis.

## Introduction

Cervical infections are uncommon disorders that represent 2–7% of all skeletal infections^1^. Pyogenic spondylitis and tuberculosis spondylitis are among the reported infections of the cervical spine. An incidence rate of 2 to 12 per 100,000 hospital admissions of cervical infection has been reported, but this incidence has doubled during the recent two decades^2,3^. Cervical infection may result from pathogens that are hematogenously transferred from a distant diseased site or directly from the adjacent locus attacking the vertebrae and intervertebral discs. The infection mainly involves the anterior column of the cervical spine and rarely attacks the posterior column. Complications including cervical kyphosis, loss of range of motion, spinal cord compression, nerve route compression, neurological deficits, and dysphagia may occur to the patient following cervical spine infections^4,5^.

Antibiotic therapy in cervical infection is advocated in the early stage of the disease and for patients without complications, while both medical and surgical treatment methods are adopted in the late stage of the disease and for patients with complications^6^. Several surgical techniques are used in the treatment of cervical spine infections including the anterior approach, posterior approach, and combined anteroposterior approach^7^.

Despite all the above surgical techniques, treatment of refractory kyphotic cervical deformity is still a challenge. We define refractory kyphosis as a change in segment Cobb angle of less than 10° from flexion to extension on dynamic X-rays^8,9^. The rapid correction of the refractory kyphotic may increase the risk of spinal cord injury and paralysis. Moreover, single-stage correction of refractory kyphosis curve subjects the patient to a high risk of implant failure as a result of high bone-screw corrective forces^10–13^. Recently the use of halo-traction in spinal surgery has shown the advantages of relaxing the soft tissues, gradual correction of kyphosis, and training the cord to acclimatize tension forces. But halo-traction has often been used during the correction of kyphoscoliosis, congenital or idiopathic scoliosis^14,15^.

To the best of our knowledge, the use of halo-traction in the treatment of refractory cervical kyphosis secondary to infections has never been reported.

The aim of this study is to investigate the role of halo-traction in the treatment of cervical infection patients associated with refractory kyphosis.

## Methods

From January 2010 to January 2020 a total of 48 patients (30 male and 18 female) with a diagnosis of cervical infections associated with refractory kyphosis were randomly reviewed from the hospital database. The patients were divided into two groups (A and B). Group A underwent preoperative halo-traction and surgery, while group B underwent surgery alone. Patients were randomly assigned to group A or B. Group B also included patients who refused traction or had contraindications for traction such as low bone mineral density, brachial plexus injury, or tight soft tissue. After approval from the institutional review board of Xiangya Hospital of Central South University, an informed consent form was obtained from each participant. And all methods were carried out in accordance with relevant guidelines and regulations of Helsinki declaration of 1964 and its amendments^16^. Data were retrieved from the medical records. The demographic data of the patients are shown in the Table 1 below.

**Table 1:** Preoperative patients dermographic data for traction group and non-traction group.

	Group A	Group B	Significant level
Gender (M/F)	14/9	16/9	P = 0.82 (χ^2^ = 0.05)
Age	53.78 ± 12.26	47.56 ± 15.49	P = 0.17 (Z = − 1.36)
NDI score (%)	46.13 ± 15.51	44.72 ± 12.35	P = 0.73 (t= 0.35)
Kyphosis Cobb angle (degree)	19.67 ± 5.63	19.20 ± 5.21	P = 0.98 (Z = − 0.02)
**ASIA**			
A	0	0	P = 0.67 (χ^2^ = 0.79)
B	3	2	
C	11	15	
D	9	8	
E	0	0	

*NDI* neck disability index score, *M* male, *F* female, *ASIA* American spinal injury association impairment scale.

### Inclusion criteria

1. The diseased lesion involving one cervical vertebral or two adjacent cervical vertebral.
2. The patients with refractory segment kyphosis as seen in X-rays.
3. Patients presented with symptoms of spinal cord or nerve root complications.
4. Patients with complete 2-year follow-up data.

### Exclusion criteria

1. Patients with other spinal abnormalities such as spinal tumor.
2. Patients with non-refractory kyphosis.
3. Patients with incomplete follow-up data.
4. Patients with huge paraspinal or spinal canal abscess

### Preoperative preparations

Before surgery, all patients underwent the examinations including: X-ray of the cervical spine (lateral dynamic and anteroposterior views), computed tomography (CT) of the cervical spine (both axial and sagittal planes), T1 and T2 weighted, magnetic resonance imaging (MRI) of the cervical spine, electrocardiogram, cardiopulmonary function, chest X-ray, liver and renal function tests, ESR, CRP and tuberculin test. Patients were given antibiotics prior to surgery according to the culture results, and some patients were already taking antibiotics at the time of admission. Additionally, the patients received liver-protective medications, treatment for hypoproteinemia, and nutritional supplementation prior to surgery.

### Halo-traction

#### For group A

**Figure 1:**
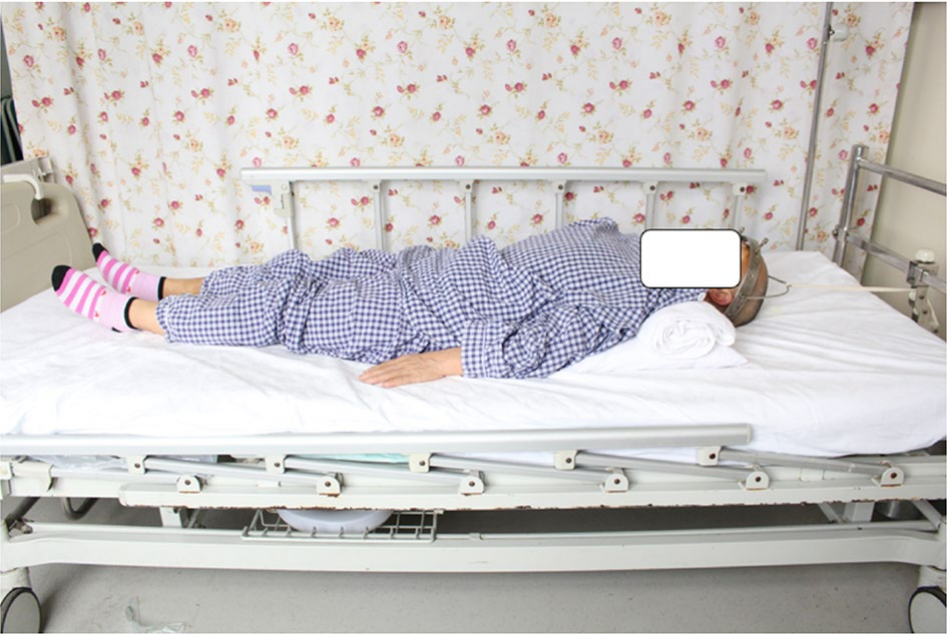
A kyphotic deformity patient with a cervical infection being treated using halo-traction.

Preoperative four pins halo-traction construct was applied at the equator of the skull, 2 pins at the anterior safe zone and other 2 at the posterior occipital bone tightened to 6–8 pounds by torque wrencher (Fig. 1). The traction started with 1kg and increased gradually each day based on individual tolerance and flexibility of kyphosis to a maximum of 1/10th of the total body weight. Our limit of traction was when the correction for kyphosis deformity was > 10^0^ degrees or did not reduce with traction. And sometimes we do stop traction at any moment if the patient develops complications. The traction time was 6.86 ± 0.87 days. During traction, the occipital neck was elevated by a pillow for about 10 cm to ensure hyperextension. A serial X-ray films were taken to determine reposition of kyphosis after traction. The pins sites were dressed daily, retightening of the pin was done every 24 h.

### Surgical method

A typical Smith–Robinson technique was used for the anterior approach to expose the cervical spine. After routine exposure, the diseased tissues, the disc and vertebral bodies were removed by curettes and longeurs. The paravertebral abscess was drained and debridement was done. Following corpectomy and decompression, the cartilage endplates of the superior and inferior vertebrae were excised and the bony endplates were preserved. Gently distraction was done using an intervertebral body spreader between the adjacent normal vertebrae to correct the kyphosis. The titanium mesh cage (TMC) contoured to fit the bony cavity was filled with allograft bone and placed in the bony endplates of the superior and inferior vertebrae. TMC was placed with the gradual distraction of the cervical spine followed by the release of the distraction. This allowed firm compression of the TMC. The anterior instrumentation, cortical lips, and the host bone all worked as buttresses to keep the TMC in place. Anterior plates were then screwed onto the nearby vertebrae. C-arm was used intraoperatively to confirm the operation site and the position of the TMC. Local administration of the antibiotics according to the culture results was done. Finally, the drainage was inserted and the incision site was closed in layers. Other tissues were taken for further histopathological examination. Neurological monitoring was done during the whole time of operation to assess both motor and sensory signals.

### Postoperative care

The drainage tube was removed when the flow was less than 30 ml/24 h. Patients continued with oral antibiotics postoperatively and were encouraged to ambulate with hard cervical collar for at least 3 months. Functional rehabilitation exercise was encouraged to all patients during early stage of recovery. Patients were asked to attend out patient clinic at 3, 6, and 12 months postoperatively and then once a year. During follow-up radiographic images were taken, and we recorded change in kyphosis Cobb angle, quality of life improvement by NDI, neurological recovery by ASIA, ESR and CRP, complications. The results of the two groups were analyzed.

### Analysis

IBM SPSS (version 22) was used for statistical analysis. Independent sample t-test was used for continuous variables that follow the normal distribution and the Wilcoxon sum rank test for the variable that does not follow the normal distribution. Chi-square test was used for categorical variables. A P-value of < 0.05 was considered to be statistically significant.

## Results

In our study, mycobacterium TB was the most common cause of cervical infection (23 cases), followed by *Staphylococcus aureus* (5 cases), Aspergillus (4 cases), *Enterococcus faecalis* (4 cases), *Pseudomonas aeroginosa* (4 cases), *Streptococcus agalactiae* (2 cases), *Escherichia coli* (2 cases), *Salmonella dublin* (1 case), *Streptococcus pneumoniae* (1 case), *Viridans streptococci* (1 case) and *Mycobacteroides chelonae* (1 case).

Traction group has a better kyphosis correction rate compared to non-traction group. The traction group had better neurological improvement as assessed by ASIA scale and better improvement of quality of life by NDI than the non-traction group, although the difference was not statistically significant. Compared to the non-traction group, the traction group has fewer number of fusions (Table 2). No significant differences between the two groups in terms of ESR and CRP (Bar Chart 1). 3 cases in group B underwent revision surgery.

**Table 2:** Postoperative results between traction group and non-traction group.

	Traction group (A)	Non-traction group (B)	P-value
Post-traction correction angle (degree)	24.78 ± 10.00	–	–
Post-operative correction angle (degree)	27.01 ± 11.54	18.08 ± 10.04	P = 0.01 (Z = − 2.44)
Number of fused segments	3.30 ± 0.63	3.52 ± 0.71	P = 0.35 (Z = − 0.93)
Final follow-up NDI (%)	18.74 ± 9.11	19.12 ± 7.89	P = 0.87(t=0.15)
**Final follow-up ASIA**			
A	0	0	P = 0.68(χ^2^ =0.17)
B	0	0	
C	0	0	
D	7	9	
E	16	16	

*NDI* neck disability index score, *ASIA* American spinal injury association impairment scale.

**Bar Chart 1:**
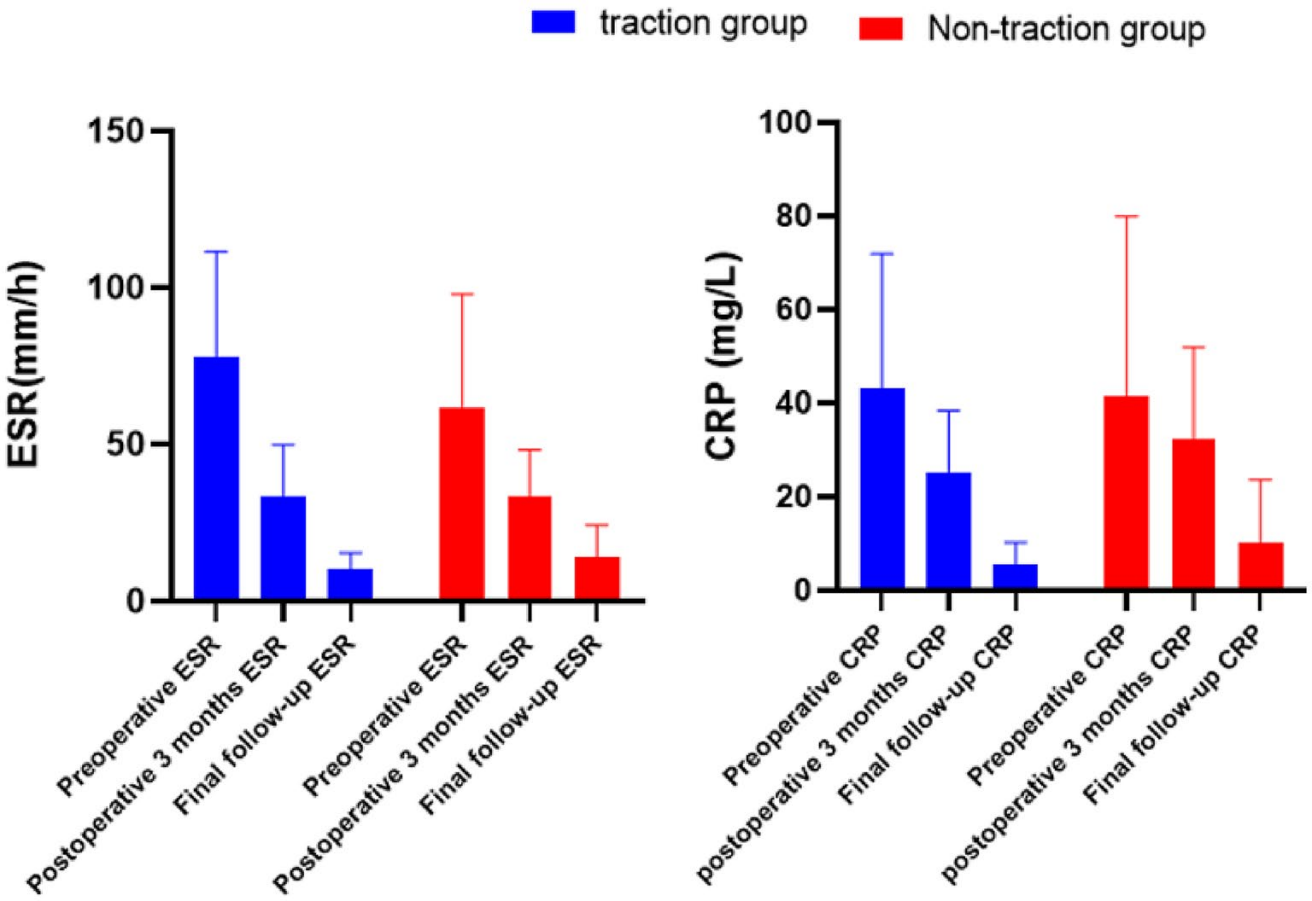
The change in ESR and CRP in traction and non-traction group.

## Discussion

**Figure 2:**
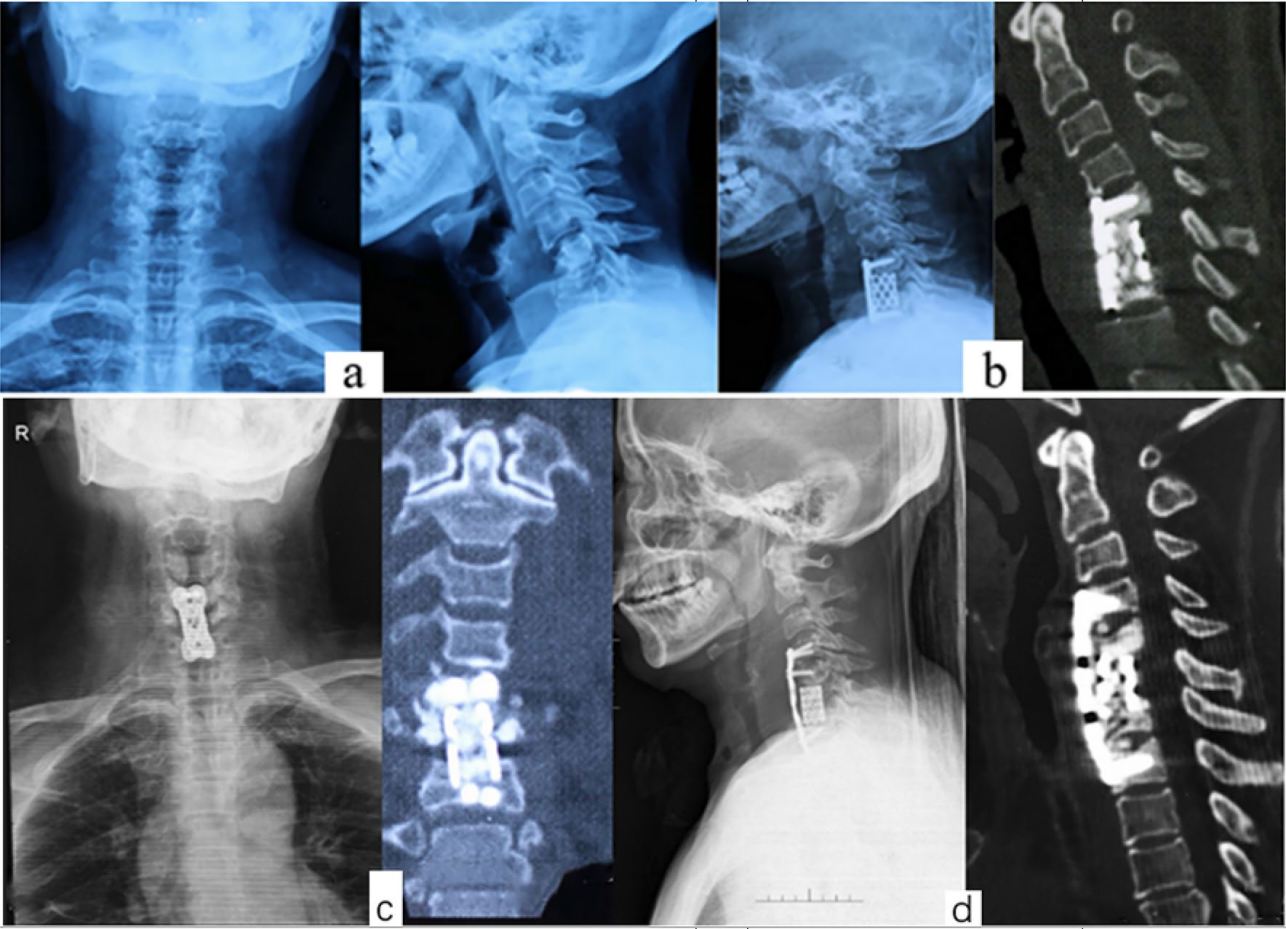
A 61 years old female with cervical TB at C5–7 and refractory kyphosis who didn’t undergo preoperative traction, (**a**) preoperative AP and lateral X-rays, no dynamic X-ray films because the patient was unable to flex or extend the neck. (**b**) Postoperative X-ray and CT, the disease lesion was cleared but the kyphosis was still there. (**c**) AP X-rays and CT-scan showing subsidence of titanium mesh, thus second operation was necessary to correct kyphosis and restore stability. (**d**) X-ray and CT-scan after the second operation. The kyphosis deformity was corrected.

**Figure 3:**
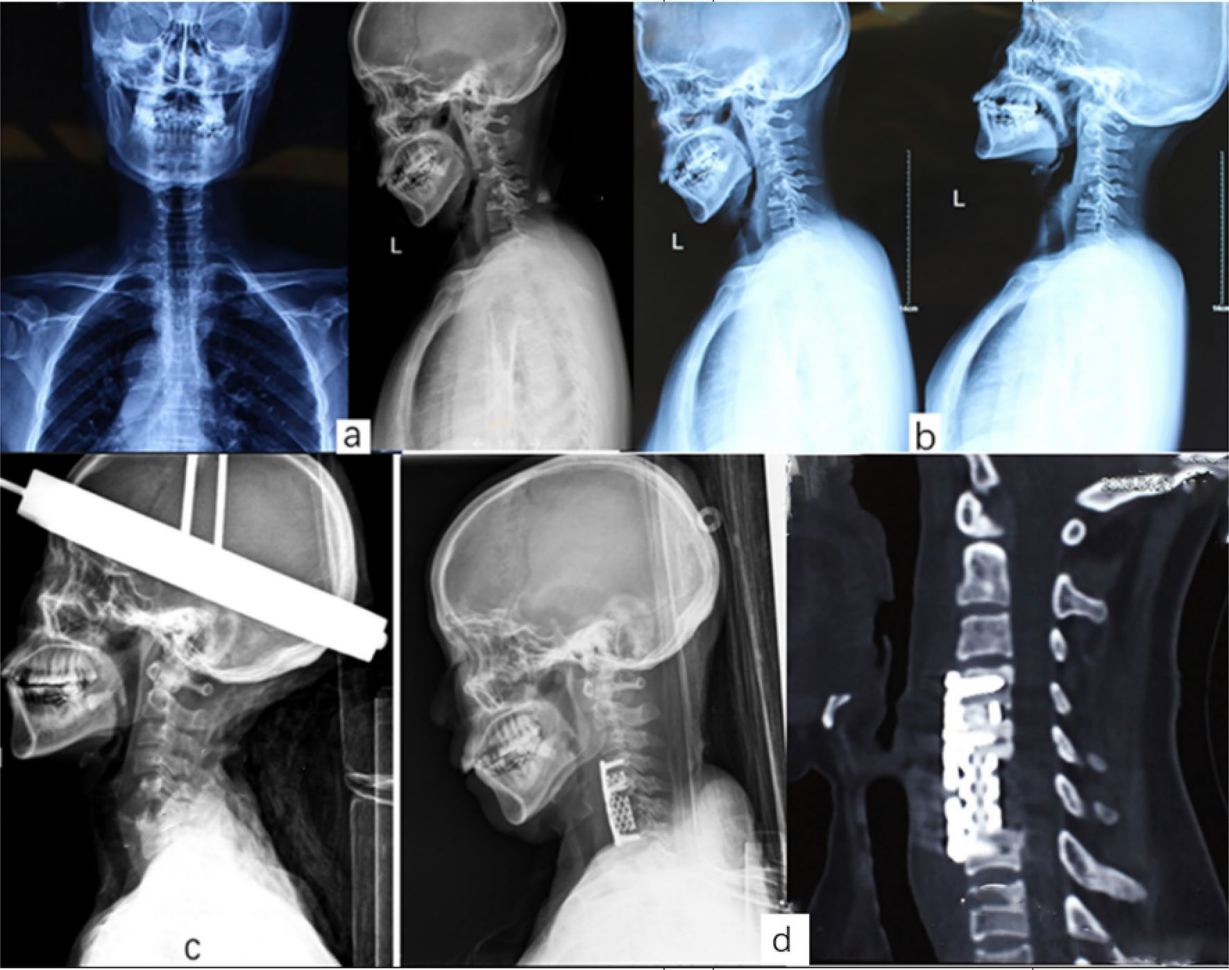
A 63 years old female with C5–6 TB infection with refractory kyphosis who underwent preoperative skull traction (**a**) preoperative AP and lateral X-ray films, (**b**) dynamic X-ray showing no obvious change in kyphosis in both flexion and extension, (**c**) after 8 days of halo-traction the cervical lordosis was recovered, (**d**) postoperative X-ray and CT-scan at final follow-up showing good bone fusion and lordosis restoration.

The lordotic curve is the natural curvature of the cervical spine, which means that the axial load lies posterior to the vertebral bodies of C2–C7. Commonly, cervical infection targets the anterior and middle column, resulting in kyphosis. With kyphosis, the weight-bearing axis slips anteriorly, placing additional strain on the anterior column. The kyphosis deformity may cause a number of consequences, including neurological deficit, functional incapacity, etc., that may require surgical intervention^17,18^. In this study we have compared kyphosis patients with cervical infection who underwent halo-traction with those who didn’t undergo traction. We found that the traction group has superior kyphosis correction compared to non-traction group. Zeng et al.^19^ in their study to investigate the role of halo-traction to cervical tubercular spondylitis patients with kyphosis obtained a similar results. Despite their study lacked a control group. This may be consistent with the idea that additional serial weight in halo-traction relaxes the soft tissue, gradually corrects the kyphosis deformity, and trains the spinal cord to adapt tension forces^20^. In our cases, we’ve noticed another very interesting thing about traction: when a patient’s pre-operative flexibility, as seen on a dynamic X-ray, is very poor, the patient must undergo traction, or the risk of failure after surgery is high (Figs. 2, 3). As a results 3 patients in non-traction group were reoperated secondary to implant failure and poor kyphosis correction. This may be explained by the reason that single-stage correction of refractory kyphosis curve subject the patient to a high risk of implant failure secondary to high bone-screw corrective forces^10,21^.

The traction group had a better neurological recovery than non-traction group, despite the fact that the difference was not statistically significant. Zhang et al.^22^ in their retrospective study to evaluate the role of preoperative halo-traction in neurofibromatosis patients with cervical kyphosis reported a similar finding of neurological recovery. The possible explanation for the neurological deficit brought on by cervical kyphosis is that the apical vertebrae and the neighbouring intervertebral disc are being compressed. Traction can extend the intervertebral space and bring the intervertebral disk back to a medium level. In addition, cervical infection primarily attacks the anterior column, Smith–Robinson approach ensures full removal of the diseased lesion in the anterior column.

The patient’s self-reported quality of life in the traction group, as measured by the NDI score at the last follow-up, was slightly greater than that in the non-traction group. Rulleau et al.^23^ reported a better improvement in neck pain and quality of life in patients who underwent strong neck traction relative to control. Moreover, during the final follow-up, the authors noted a better improvement of radicular pain in the traction group relative to the control group. Also, the number of fused segments were few in the traction group relative to the non-traction group. Even though this difference was not statistically significant between the groups. We think this is perhaps because our sample size was smaller, and no previous study has investigated the number of fusions between traction and non-traction groups to be compared. Regarding the reoperation rate, 3 patients in the non-traction group underwent the second surgery through posterior approach as a result of a failure to restore kyphosis and the persistence of symptoms. Among the complications reported by researchers, non has reported revision surgery in traction cases and worse most studies lack a control group^10,24,25^.

Patients with cervical infection associated with refractory kyphosis deformities are difficult to treat. Rapid correction of refractory kyphotic curve increases the danger of spinal cord injury and paralysis. Moreover single-stage correction of refractory curves may expose the patient at a risk of implant failure secondary to high bone-screw corrective forces. Especially in infectious vertebra where most of the anterior and middle column are destroyed by the disease lesion, reducing the anatomy for adequate hardware purchase to maintain correction^11,19^. The main objective of preoperative traction is to reduce major neurological risk while effectively obtaining correction at a regulated safe ways. In our spine department, halo-traction is part of normal routine when dealing with rigid kyphosis or other spinal deformities such as scoliosis and kyphoscoliosis.

In this study, patients who underwent traction correction was observed during the first week of halo-traction. But when we are dealing with other rigid spinal abnormalities like kyphosis scoliosis or kyphoscoliosis duration of halo-traction may be prolonged to 5 weeks and even more.

Halo-traction is sometime associated with risks. Pin loosening, superficial and deep pin-site infection, nerve injury and dura penetration, pin embarrassment and cosmetic scars have been reported^26–28^. Our traction time was short, the halo-pins were placed on the anterior safe zone and posterior occipital bone and further tightened with torque wrencher to 6–8 pounds. The pin site were dressed daily and checked for any loosening. We increased the traction weight gradually according to the patient torelance and plasticity of the kyphosis. After adhering to the above practices non of our patients developed the above mentioned complications. However we still admit that the risk during traction must not be ignored especially when dealing with patients with osteoporosis. Foristance cheung et al.^29^ reported iatrotrogenic fracture of the cervical spine during traction in 80 years old patient. Moreover the radiological film of that patient shows a severe osteoporotic bone. This still remind us about the role of the bone quality during traction.

Several limitations of this study shall be addressed. The study is retrospective in nature with some data missing, and the cervical infection cases are rare and no more articles to support our results. Also, the infectious organism were not the same in all patients. Moreover the study was conducted in a single center.

Despite cervical infection patients with kyphosis undergoing traction being rare, we still call upon further study from multiple centres and if possible randomized controlled study to further verify our findings.

## Conclusion

Preoperative halo-traction followed by surgery is superior in kyphosis correction and is associated with less complication when used in the treatment of patients with cervical infections with refractory kyphosis deformity compared to surgery alone.

## Supplementary Information

Supplementary Information.

## Data Availability

All data used in the analysis of this study are included as Additional File 1.
